# The Charter T. S. M. A.

**Published:** 1889-07

**Authors:** 


### THE CHARTER.

We have cause for congratulation that the Association is now incorporated; it has now "official status". The charter is the first step towards recognition which has been secured, and will doubtless lead to important results. Time will come—it may not be in one day—but those who come after us will see it, when the *licensing of physicians to practice medicine will be done by a board appointed by the State Association*, and in the future the Boards of Health will be administered, as is now done in Alabama, by this body. The change will be slow—but it is inevitable. Every indication points to the inevitable separation of the *licensing* from the *teaching bodies*, and when that is done it will be relegated to the proper source, the organized profession of each State.

The adoption of the resolution to apply for a charter was unanimous by the Assciation, though we learn, and with some surprise, that there are thinking members who believe we have made a mistake in incorporating. We will not attempt here to give their reasons; but it seems to us that it is a most judicious step, looked at from any standpoint. It frees the members, individually, from pecuniary responsibility, in case of suit for damages, for instance. We have been informed that where an expelled member, for example, or one who feels that he has been damaged by any action of the Association, could, under the old status, recover from the individual members; that in bringing action, writs would be served on each member, and in case of judgment, execution would issue against the property of any member found in possession of sufficient to satisfy it. A charter changes this, and makes the Association, collectively, responsible, and in case of judgment for damages, nothing would be liable but the assets of the body.

We would be glad to have the opinion of some of the "fathers" on this subject, for publication.

